# Laboratory testing of low concentration (<1 ppm) of copper to prolong mosquito pupation and adult emergence time: An alternative method to delay mosquito life cycle

**DOI:** 10.1371/journal.pone.0226859

**Published:** 2020-05-21

**Authors:** Mohamad Reza, Cimi Ilmiawati

**Affiliations:** 1 Department of Biology, Faculty of Medicine, Andalas University, Padang, West Sumatra, Indonesia; 2 Division of Environmental Toxicology, Department of Pharmacology, Faculty of Medicine, Andalas University, Padang, West Sumatra, Indonesia; Al-Azhar University, EGYPT

## Abstract

**Introduction:**

Larvicide application in ovitrap is one of the currently available methods used in mosquito eradication campaign. We previously reported that copper in liquid form is a promising candidate due to its potent larvicide properties in a laboratory setting and in the field. In the field study, several larvae survived in outdoor ovitrap due to the dilution of copper concentration by rainwater. The surviving larvae were smaller and less motile. This led our interest to study the effect of a sub-lethal dose of copper in ovitrap on mosquito larval development, pupation time and lifespan in the adult stage.

**Methods:**

First instar larvae of *Aedes albopictus*, *Anopheles stephensi*, and *Culex pipiens* were put in water containing 0.15 ppm, 0.30 ppm, and 0.60 ppm of copper. The surviving larvae, the emerging pupae, and adult mosquitoes were observed and counted every 24-hour and statistically analyzed by t-test or Mann-Whitney *U* test. Inter-species difference in response to different concentration of copper were also analyzed.

**Results:**

Copper showed a potent larvicide effect at 0.60 ppm concentration. Prolonged pupation time and a lower number of adult mosquitoes were observed at 0.15 ppm concentration. Copper exposure did not affect adult mosquitoes’ lifespan. *Culex pipiens* was the most susceptible species to copper exposure.

**Conclusion:**

This study demonstrates the efficacy of copper at <1 ppm to kill mosquito larvae and to prolong pupation and adult emergence time. Utilization of copper at a low concentration is cost-efficient in the public health setting and remains an open option as an environmentally safe vector control strategy.

## Introduction

Mosquitoes are causing millions of deaths every year worldwide. More than half of the world’s populations live in areas where mosquitoes are present as vectors and transmit malaria, dengue, Zika, Chikungunya, yellow fever and other diseases [1]. Mosquito-borne diseases are causing an enormous burden on the public health system in many countries with limited financial and human resources. Despite being considered as the most conceivable method to eradicate mosquito-borne diseases [1], global funding for malaria vector control is far below the budget endowed for exploring cures and vaccines for these diseases (US$ 56 *vs* 408 million in 2018) [2]. Lack of funding is considered as one of the challenges in achieving and sustaining malaria control [3].

Apart from the financial challenge, vector control is facing an increase in mosquito resistance to insecticides [4]. Therefore, there is a need to develop novel methods for vector control, particularly practical and economical ones, to be combined with the available methods in integrated vector management. Larval source management (LSM) is part of integrated vector control using strategies to modify or manipulate water bodies as the potential larval habitats of mosquitoes to prevent maturation of mosquito developmental stages. LSM strategies also include the introduction of larvicide (chemical and biological agents) and larvivorous fish (natural predator) into larval habitats [5–7].

Previous studies have proposed the utility of using metallic [8, 9] and liquid [5, 10, 11] copper (Cu) as a potential and affordable larvicide [7]. Copper is effective as a mosquito larvicide at a concentration below 2 ppm [9], the threshold value deemed safe for human drinking water [12], thus making copper a potential candidate for use in the public health setting.

A laboratory study using metallic copper showed that 10 g/L and 20 g/L can hinder *Aedes albopictus* larval development and induce high larval mortality (8). A more recent effort reported that metallic copper spray lead to 100% mortality of *Aedes* larvae tested in 14 days (9). Our previous findings on the properties of copper to kill mosquito larvae [10] and laboratory testing on the effect of liquid copper at a concentration of 10 ppm on three species of mosquito larvae [11] prompted us to perform a field test in a malaria and dengue-endemic area of West Sumatra, Indonesia. Despite confirming the larvicide effect of liquid copper [7], we observed that rainwater diluted the copper in outdoor ovitrap and some larvae survived. However, the survived larvae appeared to be less motile and were smaller in size compared to larvae unexposed to copper. This observation intrigued us to determine whether the survived larvae capable to mature into adulthood. Therefore, we performed a laboratory test of sub-lethal concentrations of copper exposure to mosquito larvae of various species to verify its effect on mosquito development and to compare inter-species susceptibility to copper exposure.

## Materials and methods

### Ethical approval

All mouse procedures were approved by the Institutional Animal Experiment Committee of Jichi Medical University. This research was approved by the ethics committee of the Faculty of Medicine, Andalas University.

### Animals

*Anopheles stephensi* SDA500 strain, was maintained in 26°C, 60–80% relative humidity, and a 13-h/11-h light/dark cycle at Jichi Medical University [13]. Adults were fed on a filter paper soaked with 5% fructose (Nacalai Tesques, Kyoto, Japan). Female BALB/c mice were purchased from SLC (Shizuoka, Japan). Females mosquitoes were allowed to feed on anesthetized mice on day five after emergence [14]. Three days later, an oviposition dish was placed in a cage containing gravid females. *Aedes albopictus* and *Culex pipiens* eggs were obtained commercially and the first instar larvae were utilized in the experiment. The eggs were laid on a filter paper placed on a 12 x 20 cm hatching tray containing 500 mL of water. Larvae were fed with fish food, Hikari (Kyorin, Himeji, Japan) twice daily.

### Preparation of CuSO_4_ solution

The stock solution was made by dissolving 16.0 g of CuSO_4_ powder into up to 1000 mL of dechlorinated filtered water, to have a 100 mM CuSO_4_ solution (6.35 g of copper in 1000 mL). Then, we prepared a 10.0 ppm copper solution (= 10 mg copper/L) by diluting 1.0 mL of the stock solution into 634 mL of filtered water. The 0.60 ppm copper solution was prepared by diluting 60 mL of the 10.0 ppm solution with 940 mL of filtered water. Then, 0.30 and 0.15 ppm copper solution were obtained by serial dilution. The copper level was confirmed using a copper measuring device (Hanna Instruments, Tokyo, Japan) and Z-5010 Polarized Zeeman flame atomic absorption spectrophotometer (Hitachi Ltd, Tokyo, Japan).

### Copper exposure and observation of larvae, pupae, and adult mosquitoes

First instar larvae of *Ae*. *albopictus*, *An*. *stephensi*, and *Cx*. *pipiens* were used in this experiment. Forty larvae of each species were put in a container of control, 0.15, 0.30, and 0.60 ppm copper solution. Larvae were allowed to hatch and were observed every 24-hours until pupae emerged. Then, pupae were observed every 24-hours until becoming adult mosquitoes. Adult mosquitoes were observed until day 22 of the experiment. The experiment was performed in three replicates for each species. The number of larvae, pupae, and adult mosquitoes in each container were counted and statistically analyzed.

### Statistical analysis

The effect of different concentration of copper on the number of larvae, pupae, and adult mosquitoes of each species, and the assessment of inter-species difference in response to copper were analyzed by using two tail t-test or Mann-Whitney *U* test compared to control. All statistical analysis was performed by IBM SPSS ver. 25 (IBM, Inc.) and the significance level was considered at p<0.05.

## Results

**Fig 1:**
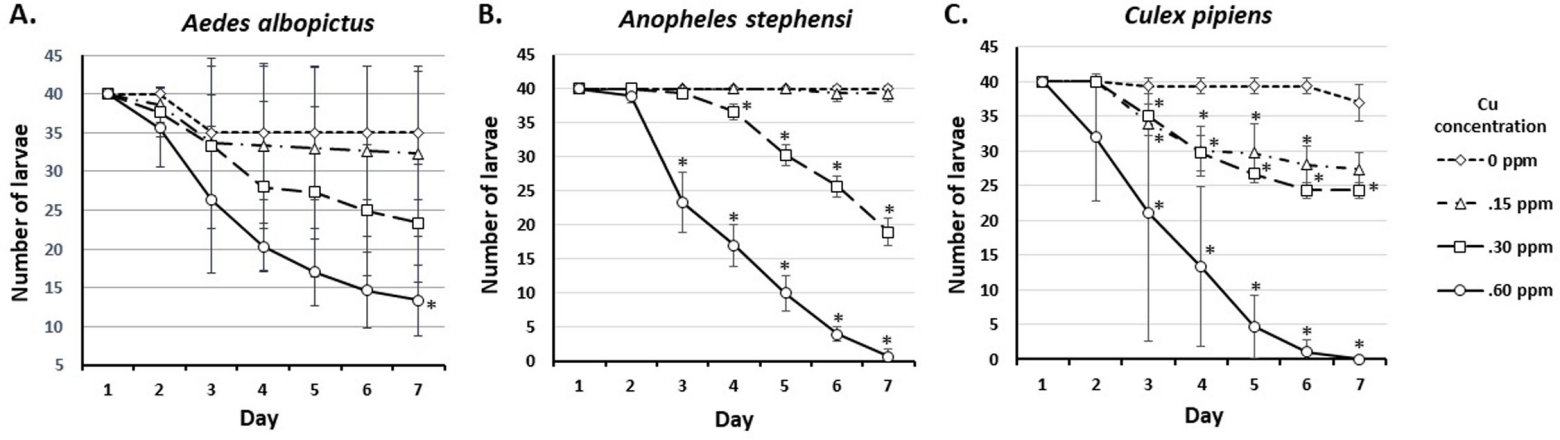
The observed mortality of *Ae*. *albopictus*, *An*. *stephensi*, and *Cx*. *pipiens* larvae on exposure to 0.15, 0.30, and 0.60 ppm of Cu. *significantly different from control, p<0.05 (t-test or Mann-Whitney U test).

To determine the effect of very low concentrations of copper on mosquito larval mortality, larvae of *Ae*. *albopictus*, *An*. *stephensi* and *Cx*. *pipiens* were exposed to 0.15, 0.30 and 0.60 ppm of copper and were observed daily. Larvae of *Ae*. *stephensi* and *Cx*. *pipiens* exposed to 0.60 ppm of copper showed 100% mortality within a week, while a small number of *Ae*. *albopictus* larvae survived. Larvae from three species roughly showed 50% mortality when exposed to 0.30 ppm of copper for seven days. Copper at 0.15 ppm had a statistically significant effect on *Cx*. *pipiens* larval mortality compared to control, started on the third day of exposure (**Fig 1**).

**Fig 2:**
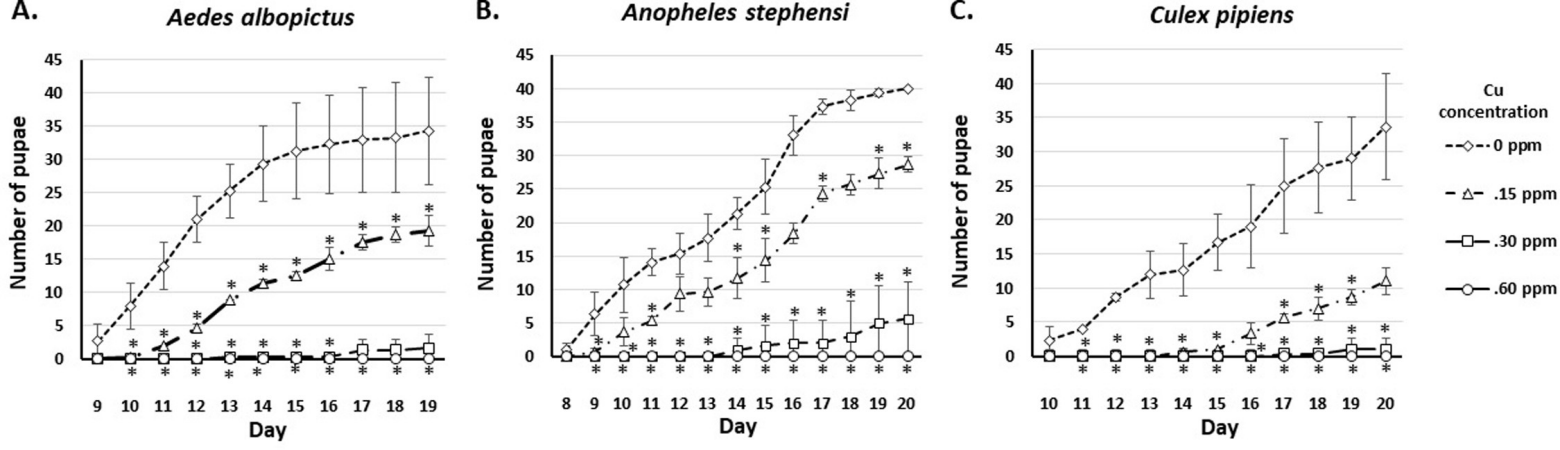
The number of pupae of *Ae*. *albopictus*, *An*. *stephensi*, and *Cx*. *pipiens* on exposure to 0.15, 0.30, and 0.60 ppm of Cu. *significantly different from control, p<0.05 (Mann-Whitney U test).

To figure out the effect of very low concentrations of copper exposure on pupation time, the number of emerging pupae from the three species were counted. Pupae started to emerge at day eight to 10, depending on the mosquito species. Surviving larvae exposed to 0.15 and 0.30 ppm of copper showed prolonged pupation time in all species observed compared to the control group. We also observed that the surviving larvae of *Ae*. *albopictus* on 0.60 ppm were not turned into pupae (**Fig 2**).

**Fig 3:**
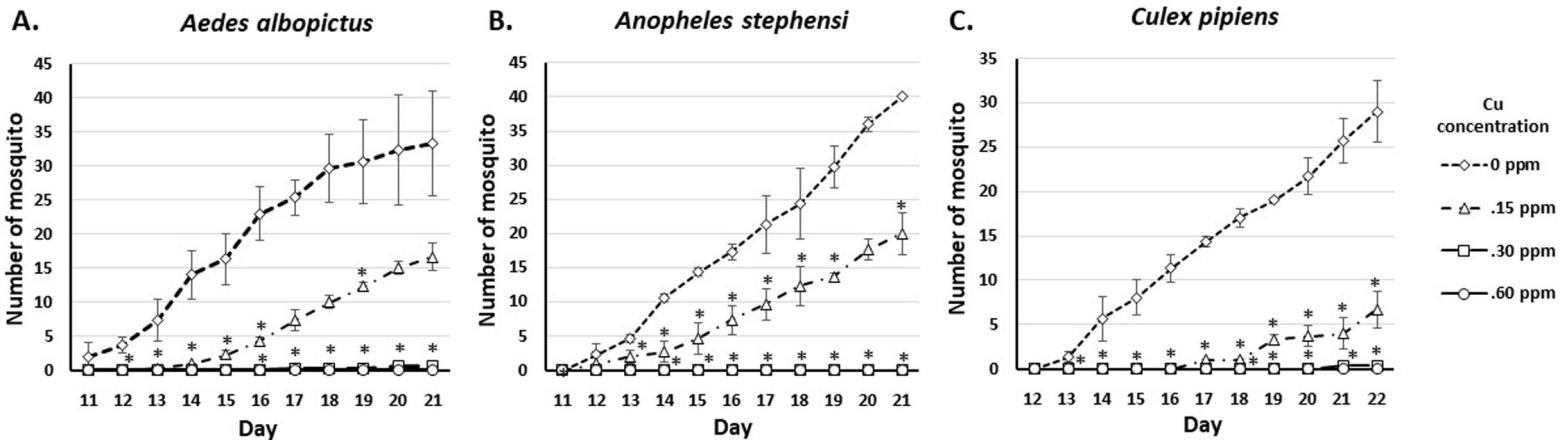
The number of *Ae*. *albopictus*, *An*. *stephensi*, and *Cx*. *pipiens* mosquito on exposure to 0.15, 0.30, and 0.60 ppm of Cu. *significantly different from control, p<0.05 (Mann-Whitney *U* test).

To determine the effect of very low concentrations of copper exposure on mosquito ability to reach adulthood, we observed and counted the number of emerging adults in the three species. Surviving pupae of all observed species exposed to 0.15 ppm of copper become adult mosquitoes, albeit at a statistically significantly lower number compared to the control group. Adult mosquito of all observed species emerged at later days compared to control and survived up to day 22 of observation. Almost none of the pupae of all observed species exposed to 0.30 ppm of copper emerged as an adult mosquito (**Fig 3**).

**Fig 4:**
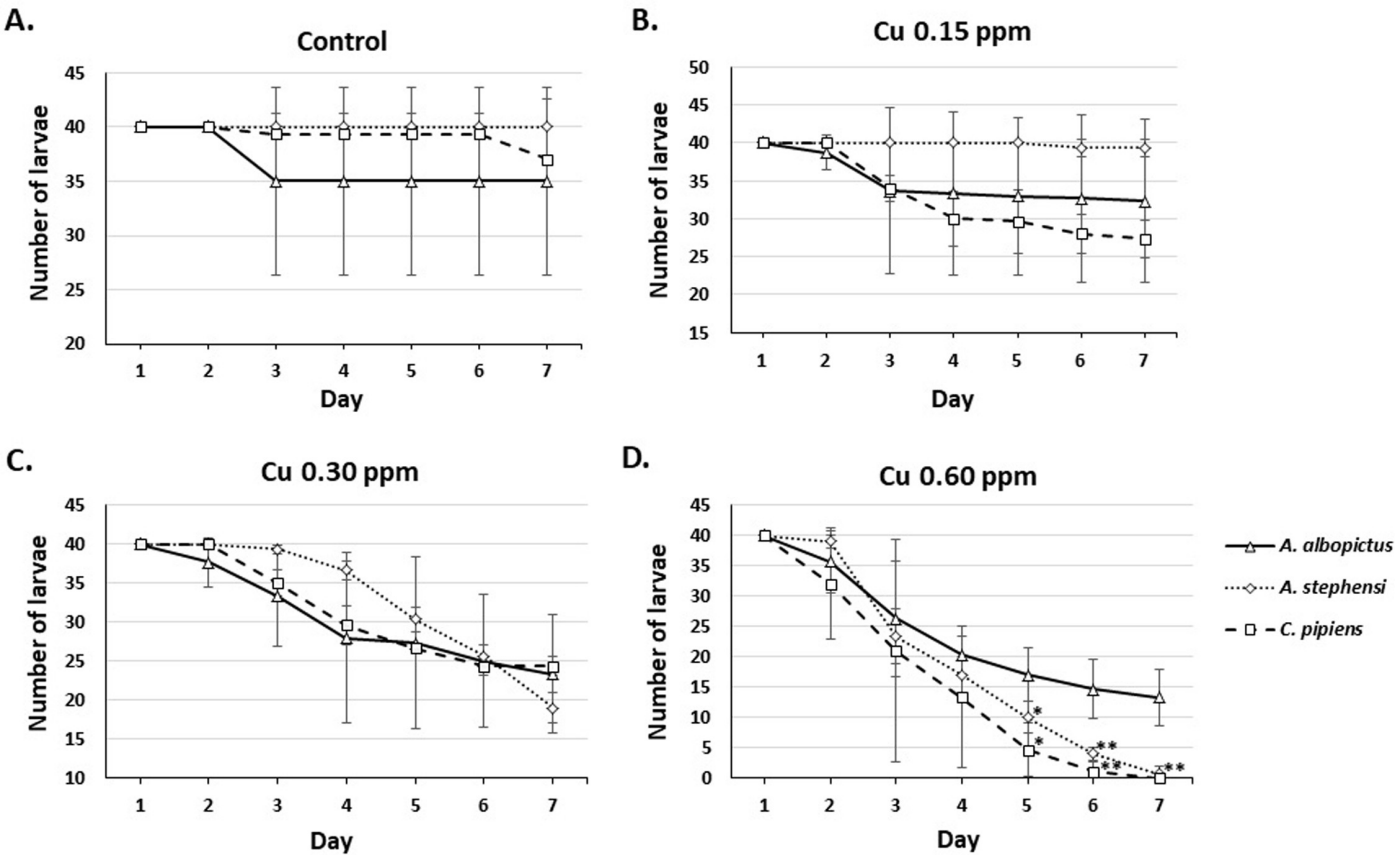
Inter-species comparison of the observed mortality of *Ae*. *albopictus*, *An*. *stephensi*, and *Cx*. *pipiens* larvae on exposure to 0.15, 0.30, and 0.60 ppm of Cu. *p<0.05; **p<0.01; compared to *Ae*. *albopictus* (t-test or Mann-Whitney *U* test).

**Fig 5:**
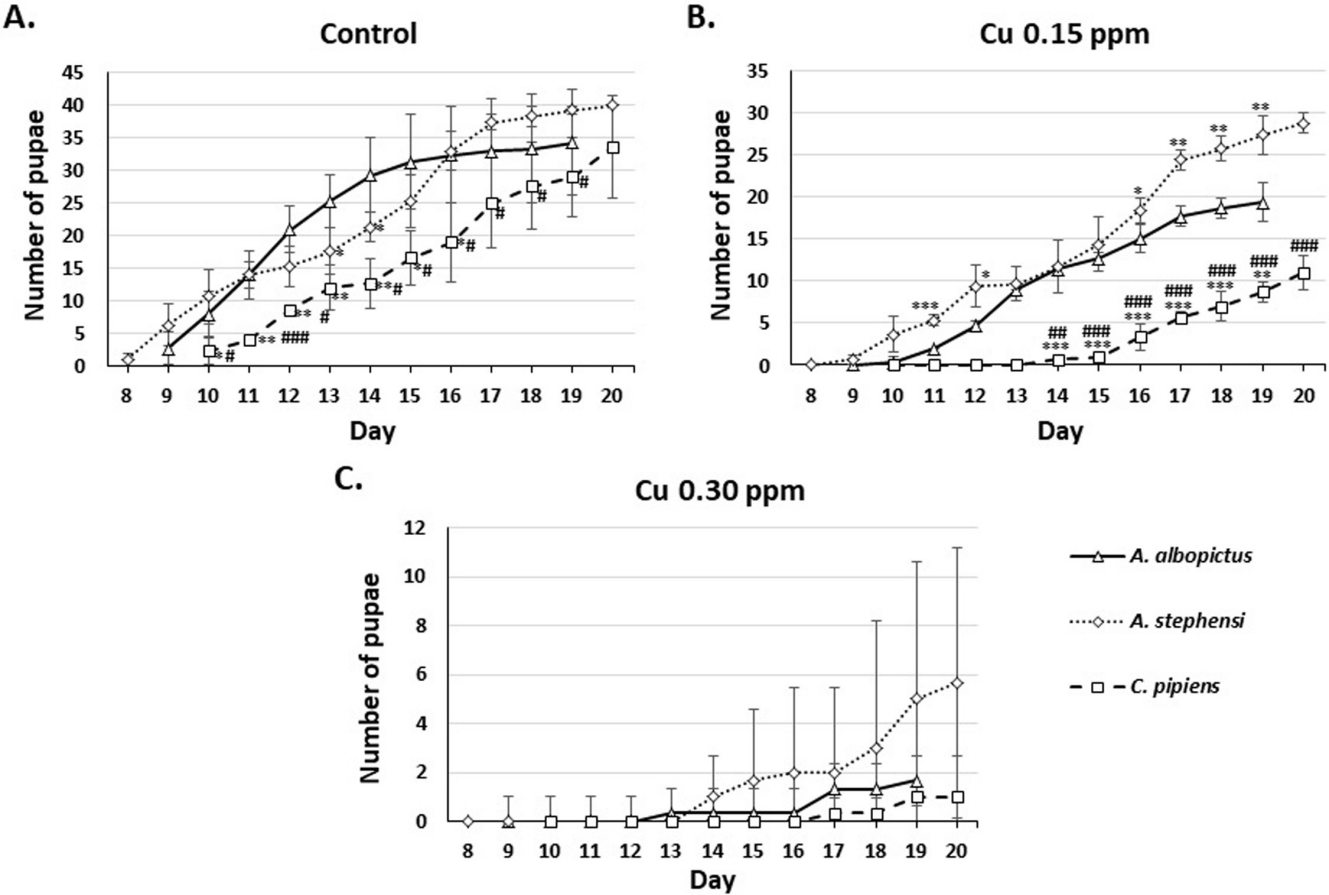
Inter-species comparison of the number of *Ae*. *albopictus*, *An*. *stephensi*, and *Cx*. *pipiens* pupae on exposure to 0.15, 0.30, and 0.60 ppm of Cu. None of the species showed pupae on exposure to 0.60 ppm. *p<0.05, **p<0.01, ***p<0.001 compared to *Ae*. *albopictus;* #p<0.05, ##p<0.01, ###p<0.001 compared to *An*. *stephensi* (t-test or Mann-Whitney *U* test).

**Fig 6:**
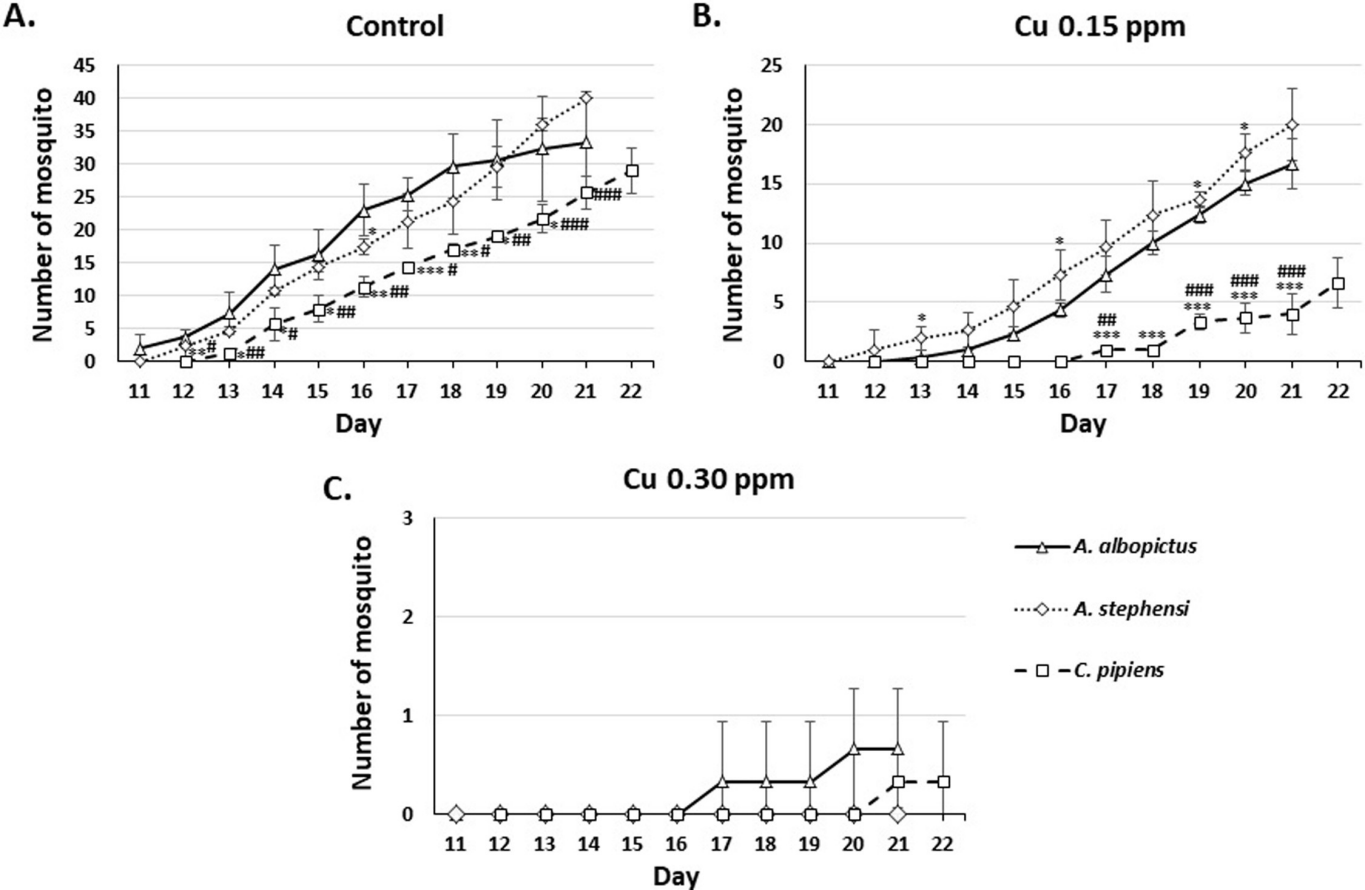
Inter-species comparison of the number of adult *Ae*. *albopictus*, *An*. *stephensi*, and *Cx*. *pipiens* mosquito on exposure to 0.15, 0.30, and 0.60 ppm of Cu. *p<0.05, **p<0.01, ***p<0.001 compared to *Ae*. *albopictus;* #p<0.05, ##p<0.01, ###p<0.001 compared to *An*. *stephensi* (t-test or Mann-Whitney *U* test).

To compare inter-species difference in response to copper exposure, we statistically analyzed the number of larvae, pupae, and adult mosquito in all species at different copper concentration and presented the result in **Figs 4–6**. **Fig 4**showed that larvae of all species were relatively similar in susceptibility at 0.15 (**Fig 4B**) and 0.30 ppm (**Fig 4C**) of copper. However, at 0.60 ppm, *Cx*. *pipiens* and *An*. *stephensi* were more susceptible compared to *Ae*. *albopictus*, started at day five (**Fig 4D**). *Cx*. *pipiens* and *Ae*. *albopictus* pupae (**Fig 5**) and adults (**Fig 6**) were more susceptible to 0.15 ppm of copper compared to *An*. *stephensi* (**Figs 5B and 6B,** respectively). At 0.30 ppm, pupae and adults of all species demonstrated similar susceptibility (**Figs 5C and 6C,** respectively).

## Discussion

Our result confirms that copper exposure at a low concentration of 0.60 ppm lead to a high mosquito larval mortality rate. At a further lower concentration of 0.30 ppm, liquid copper kill half of the larvae and at 0.15 ppm, prolongs pupation time and adult emergence time compared to the control group. The number of mosquitoes that emerged afterward is also significantly lower than those in the control group.

It is noteworthy that all adults emerged in copper-treated groups and control appeared to be viable and showed no difference in term of their lifespan. It seemed that the copper effect on the survived larvae was limited to prolonging the emergence of pupae and was not carried on to the adult stage. The effect of copper on the larvae was probably mediated by its effect on the commensal bacteria in the larval midgut, causing intestinal dysfunction [15]. This hinders their ability to process food and limiting their energy provision for pupation and adult emergence. Copper ability at a very low concentration to prolong mosquito life cycle opens the possibility to utilize copper in small container habitats to jeopardize mosquito’s developmental maturation. When mosquito’s maturation time is longer, they are becoming less efficient as disease vectors.

*Aedes sp*. are known to be resistant to the insecticides used in indoor or outdoor spraying. A study conducted in Sri Lanka showed that *Ae*. *aegypti* and *Ae*. *albopictus* were highly resistant to DDT and they can oviposit indoors and outdoors [16]. *Ae aegypti* is also known to be opportunistic, having the ability to adapt to changing environment, and a container breeder [17]. A study in Bangladesh reported abundant potential larval habitats for *Aedes sp*. in containers or jars spread around the city [18]. The insecticide resistance and opportunistic behavior of mosquito requires an alternative approach in vector control.

The US EPA has recommended 1.3 ppm as a safe limit of copper concentration in the drinking water [19], thus making the application of 0.15 ppm of copper as an environmentally safe option for vector control. This purpose may be served by using copper in small container habitats in targeted areas protected from rainfall to avoid copper dilution.

The general objective of mosquito management is to eradicate mosquito as disease vector through integrated efforts at a personal and population-wide level [1]. Considering that those at risk of contracting mosquito-borne diseases reside in poor communities of developing countries, a cost-efficient method is preferable for mosquito control. In Indonesia, the retail price of 1000 g of CuSO_4_ is about 3 US dollar. To prepare 100 mM stock solution, we need 16 g of CuSO4, which costs about 5 cents. This relatively low cost supports copper use for larval control.

Larval stage, particularly the first instar, is the most vulnerable phase in mosquito life cycle, rendering this stage easier to manipulate. Using copper at an environmentally safe concentration that is proven to kill mosquito larvae, prolong pupation time and delay adult emergence, as we demonstrated in this study, is an open option for public health vector-control strategies.
